# The evolution of capture myopathy in hooved mammals: a model for human stress cardiomyopathy?

**DOI:** 10.1093/emph/eov015

**Published:** 2015-07-20

**Authors:** Daniel T. Blumstein, Janet Buckner, Sajan Shah, Shane Patel, Michael E. Alfaro, Barbara Natterson-Horowitz

**Affiliations:** ^1^Department of Ecology and Evolutionary Biology, University of California, 621 Charles E. Young Drive South, Los Angeles, CA 90095-1606, USA;; ^2^David Geffen School of Medicine at UCLA, Division of Cardiology, 650 Charles E. Young Drive South, A2-237, Los Angeles, CA 90095, USA

**Keywords:** evolutionary epidemiology, capture myopathy, Takotsubo Syndrome, comparative medicine

## Abstract

**Background and objectives**: Capture myopathy (CM) syndromes in wildlife may be a model for human stress cardiomyopathy, including Takotsubo cardiomyopathy. Emotional stress or grief may trigger heart attack-like symptoms, and occasionally, sudden death in some humans. Similarly, wildlife exposed to predatory stresses, chase, or capture occasionally results in sudden death. To better understand the nature of vulnerability to stress-induced sudden death, we studied cases of CM in hooved mammals—ungulates—and hypothesized that CM would be associated with a syndrome of longevity-related traits.

**Methodology**: We reconstructed the evolution of CM in ungulates then determined how a set of life history traits explained variation in the likelihood that CM was reported.

**Results**: CM is broadly reported, but not in all genera, and phylogenetic analyses suggest that it is an evolutionarily labile trait. We found that the following traits were significantly associated with reports of CM: greater brain mass, faster maximum running speed, greater minimum group size and greater maximum longevity.

**Conclusions and implications**: CM may be an unavoidable consequence of adaptations to reduce predation risk that include increased running speed, sociality and having larger brains. Moreover, longer-lived species seem to be more likely to be susceptible to CM. Exploring variable susceptibility to CM highlights the evolutionary origins of the disorder, potential basic mechanisms that underlie vulnerability to the phenomenon, and the potential for reduction of risk through modification of life history trajectory.

## INTRODUCTION

Capture myopathy (CM) in wildlife may be a model for stress cardiomyopathy (e.g. Takotsubo Syndrome, Broken-Heart Syndrome) in humans [1]. Peracute CM (a sub-type of CM in which symptoms emerge soon after exposure to the stressor) in particular, shares many features with Takotsubo Syndrome/Broken-Heart Syndrome in humans. CM syndromes have been broadly described and are varied in nature. CM syndromes are referred to in the literature by a variety of other names including cramp, exertional myopathy, exertional rhabdomyolysis, muscular dystrophy, overstraining disease, white muscle disease, muscular necrosis, idiopathic muscular necrosis, stress myopathy and capture paresis [2]. These syndromes typically reference the systemic consequences of damage to the skeletal and cardiac muscles subsequent to the pursuit, capture, holding, handling and manipulation of an animal. The varied nature of these systemic consequences in animals including ataxia, paralysis, rhabdomyolysis and fatal metabolic perturbations have possibly obscured an important linkage between the more acute and probably cardiac peracute CM and acute stress CM in humans [1, 2].

Fear and distress, independent of chase and/or restraint are important factors in the aetiology of all forms of CM. Typical histologic lesions including small areas of necrosis and occasional capillary microthrombii within skeletal muscle and other organs are commonly reported. The diagnosis of CM is often made when these histologic findings are noted in association with a characteristic set of events. It is notable that characteristic histologic findings have been seen in free-ranging ungulates that had not been pursued [3] and in animals killed by hunters or dogs, and those hit by cars [4]. Sudden /surprise attacks by predators can cause CM without extensive chase [5]. The internal experience of fear triggers robust autonomic responses in terrified animals. The physiological effects of these responses contribute to all types of CM.

CM can be subdivided into three or four syndromes based on the time required for symptoms to appear, or by a mixture of time and the major signs and symptoms that can be observed. The three time-based syndromes are: peracute (characterized by hyperkalemia, cardiac fibrillation and death [6, 7], sub-acute (characterized by tubular nephrosis, renal failure and death [8]; and chronic (characterized by congestive heart failure, and death [8, 9]. Physical impairment such as lameness or loss of the ability to walk or fly can occur in non-lethal cases or before death in lethal cases.

CM likely can occur in any animal but it is believed to occur more frequently in mammals and birds, and particularly in terrestrial ungulates—artiodactyls (even toed ungulates such as camels, deer, oxen and pigs) and perissodactyls (odd toed ungulates such a horses, tapirs and rhinoceroses)—and in long-legged birds such as flamingos and shorebirds [10–14]. The reasons for such increased prevalence are unknown.

In a series of comparative studies, we study first the evolution of CM by reconstructing its origin in a particularly well-studied mammalian clade—ungulates. We report that susceptibility to CM is a labile trait meaning that it has evolved and been lost repeatedly over evolutionary time. Given this lability, we then examined life history correlates of reports of CM in ungulates. These species fall prey to a variety of carnivores [15] and, because predatory capture may trigger CM, we expected that they might be generally more susceptible to this syndrome [2]. For these reasons, ungulates make an appropriate biological model to study the covariates of CM. We focused on members of this clade to identify life history traits associated with susceptibility to CM.

Throughout, our goal was to identify the evolutionary roots of CM with the overarching goals of developing new model systems in which to study it as well as to identify relatively more susceptible and relatively less susceptible species.

## METHODS

### Developing the comparative database

We searched for publications that alluded to, described, and/or diagnosed capture related myopathy (initially in mammals and birds but later just on the well-studied ungulates) using Google Scholar and Scopus, using the terms ‘capture myopathy’, ‘exertional myopathy’ or ‘exertional rhabdomyolysis’. We then examined the articles listed by search engines as citing the identified articles. Identification and collection of articles ended in September 2013. Articles were included in our analysis if they identified CM in the death of animals or described the capture as the cause of death. Instances of vital injuries to the animal in the course of capture were not considered cases of CM; only those that described some physiological response to capture leading to eventual death were included.

### Studying the evolution of CM

To determine the phylogenetic distribution of reported CM cases we created presence/absence matrices for the trait ‘CM susceptibility.’ Species with reports for cases of CM were scored as susceptible to the syndrome while species with no such reports in the literature were scored as non-susceptible. We obtained phylogenetic trees from previous studies (mammals: [16]; ungulates [17]).

Dense sampling of characters within a phylogeny allows for the reconstruction of trait evolution. However, missing data can have a profound effect on ancestral state reconstruction and estimates of trait evolution. Because most species of mammals and birds have not been rigorously assessed for the presence/absence of CM, we did not attempt to estimate ancestral states for these groups. However, because many ungulate species have shared a close association with humans as either game or through domestication, we chose to perform likelihood-based ancestral state reconstruction of the ‘ungulate’ subtree of Ref. [17].

We reconstructed ancestral states in ungulates for the trait ‘CM susceptibility’ under maximum likelihood using the function Ancestral Character Estimation in the R package Analyses of Phylogenetics and Evolution (APE package—[18]). Assuming that the character data and the phylogeny is true and complete, the Ancestral Character Estimation function allows us to estimate a history of character evolution, evaluate the degree of uncertainty associated with those estimations and compare performance of different models of character evolution. We used Akaike Information Criterion (AIC) scores to compare the fit of the equal rates (ER) model, which assumes that gains and losses of CM are equal, to the all rates different (ARD) models that allows for differences in the rate of gain and loss of CM. An AIC difference of 4 or greater between the best model and the alternative was interpreted as providing substantial support for that model [19].

### Studying life history correlates of CM

For these analyses, we wished to maximize the life history variation and therefore focused on the clade of mammals known traditional ungulates—artiodactyls and perissodactyls. Amongst the mammals, this clade is relatively well studied with respect both to reports of CM as well as data on a variety of life history traits. We focused on life history traits that had been directly reported to be associated with CM, or indirectly reported to be associated with stress or longevity. We included longevity because CM causes death, so variables associated with lifespan may reflect the susceptibility to mortality-inducing events, like CM.

Body size, which itself is a life history trait, also has a profound influence on many life history traits [20]. Moreover, long-legged birds have been directly reported to be more susceptible to CM [21], and body mass may influence locomotor ability [22] and hence escape ability [23]. For these reasons, we included both body mass and body length as traits.

Allocation of resources to escape is a life history trait [24]. The physiology of flight in humans characterized by adrenergic stimulation, a system also activated by stress. When predators chase prey, prey are likely to be physiologically stressed while fleeing [25]. Thus, we suspect there could be a relationship between greater physiological stress levels and maximum running speed (which reflects a need to flee). For this reason, we included maximum running speed as one of our variables.

Predators select for other traits as well. By living in larger groups, individuals may dilute predation risk [26], but living in larger groups may itself be socially and physiologically stressful [27]. However, such stress-related effects are not uniformly found [27]. Nonetheless, group size is a trait that might be related to both stress and longevity, and thus we wished to understand its effects on the propensity to be susceptible to CM.

Animals group to reduce predation risk [28], and evidence suggests that brain size increases both in response to increased sociality (e.g. [29]) as well as increased predation risk (e.g. [30]). Allocation of energy to brain production is costly and brain mass is associated with longevity [31]. While relatively large-brained animals may live longer, brain size, nevertheless might be associated with vulnerability to CM.

The age of weaning is a life history trait that should form a syndrome with other traits because it is often associated with longevity [32], and therefore may be associated with susceptibility to CM. Additionally, at the intraspecific level, greater anxiety in stressful situations is associated with earlier weaning dates in male Wistar rats [33].

West and Heard [34] noted that CM was more prevalent both in very young, or very old, animals. Maximum longevity is a trait that has been reported for many species, thus, we asked whether it explained some variation in CM.

There are associations between pregnancy and CM [34]. We hypothesize that because longer gestation periods, increased litter sizes and increased number of litters per year are components that result in longer and/or more pregnancies, species with these characteristics will be more likely to have reports of CM.

We gathered life history data for all species of ungulates listed in the Supplementary Material describing presence or absence of CM: 225 species were terrestrial, and two species lived in both the water and on land. We searched for data for litter size, litters per year and species longevity for each of these 227 species [35]. We used the *CRC Handbook of Mammalian Body Masses* to obtain data for the minimum and maximum body masses of each species [36]. The handbook typically contained data for multiple individuals for each species. Thus, we recorded only the individuals with the lowest and highest body masses as the minimum and maximum body masses, respectively, for each species. We then calculated an average of these two values to obtain the midpoint body mass for each. We obtained data for gestation period, weaning period, sexual maturity of males, sexual maturity of females, minimum group size, maximum group size, minimum body length and maximum body length from Ultimate Ungulates, a curated website [37]. We calculated the midpoint body lengths from average of the minimum and maximum body lengths. We subsequently found missing data values for these traits from the *Encyclopedia of Life* and the *Animal Diversity Web* [38]. We compiled data on maximum running speed from previous studies [39, 40]. Finally, we acquired brain mass data from Pérez-Barbería [41, 42].

We fitted type-III logistic regressions using SPSS 22.0 [43], where CM was the dependent variable and each life history trait was an independent variable. Rather than doing a multiple logistic regression, we elected to fit a series of univariate models because sample sizes varied across species for available life history traits and we wanted the best evidence for each potential relationship.

Because species values are not generally phylogenetically independent [44], we fitted a phylogenetically corrected logistic regression using the R package phylolm [45] and report the phyloglm coefficients and alpha values (an estimate of the phylogenetic correlation).

## RESULTS

### The evolution of CM

**Figure 1.**
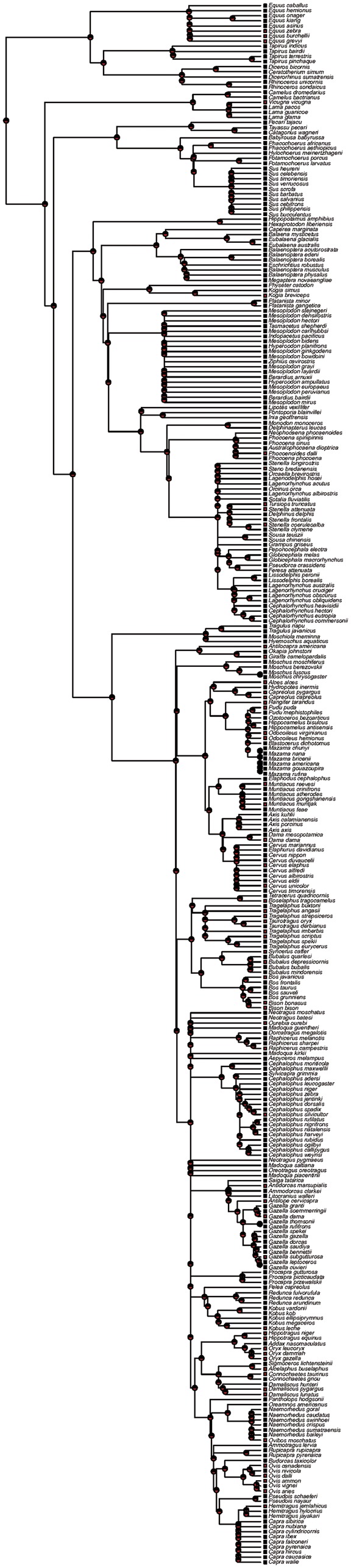
The evolution of CM in ungulates. Maximum likelihood reconstruction of the evolution of CM in the Bininda-Emonds *et al.* [17] ungulate tree under the well-supported ARD model of evolution (see text for details). Red squares are species in which CM has been reported. The amount of red in the circles illustrates the likelihood that CM was present at an ancestral node

Our ancestral state inferences are highly sensitive to the method of reporting missing data. This is seen by examining the results of the different reconstructions that varied the relative rates of loss and gain of CM. The ARD model fit the data substantially better than the ER model (AIC = 298.65, ΔAIC = 51.84). Under ARD, where the rate of gaining CM was twice as fast as the rate of losing CM, our data show an equal likelihood for presence or absence of the trait at virtually all internal nodes (Fig. 1). Under this scenario, our ability to infer the ancestral state of medium and deep internal nodes is limited, because the observed distribution of states could be explained by relatively recent or more ancient gains of the state. These findings suggest that the trait is somewhat evolutionarily labile.

### Life history correlates of CM

We present summary statistics for both logistic regression models and phylogenetically corrected phyloglm models in Table 1. Phylogenetic alpha values were often low, suggesting that these life history traits are conserved across species. Both analyses revealed that species that run faster are more likely to be reported as having capture mypopthy. The phyloglm results further suggest that species with larger brains, those that live longer, and those found in larger minimum group sizes are more likely to be reported as being susceptible to CM. Because we view these analyses as exploratory, we do not report values corrected for multiple comparisons. However, none of the analyses was significant (*P* < 0.05) after correcting for false discovery rate (the bold values were ∼*P* = 0.1), and none was significant with an even more conservative Bonferroni-corrected *P*-critical value set to 0.0038.

**Table 1.** Summary of bivariate analyses

	*N*	Logistic regression coefficient	Logistic regression *P*-value	Phyloglm coefficient	Pyloglm *P*-value	Alpha
Brain mass	97	2.425	**0.024**	2.259	**0.035**	0.427
Max running speed	39	0.043	**0.026**	0.041	**0.030**	0.012
Litter size	176	−0.640	0.138	−0.581	0.149	0.642
Gestation period	203	0.051	0.292	−0.052	0.331	0.476
Min group size	136	0.035	0.253	0.092	**0.034**	0.012
Max group size	130	0.000	0.279	0.000	0.286	0.188
Mid body mass	106	0.000	0.781	−0.001	0.148	0.012
Sexual maturity female	189	0.010	0.892	0.000	0.994	0.521
Sexual maturity male	179	−0.059	0.481	−0.139	0.132	0.652
Weaning age	159	−0.005	0.915	−0.003	0.942	0.608
Max longevity	158	0.006	0.751	0.049	**0.025**	0.012
Mid body length	191	0.001	0.200	0.001	0.214	0.012
Litters per year	113	0.142	0.794	0.144	0.792	0.437

Coefficients and *P*-values from logistic regression and phylogenetic logistic regression models*. N* is the number of species with sufficient data to calculate a given analysis. Phyloglm coefficients and *P*-values are calculated from the phylogenetic GLM package phyloglm. Alpha is the phylogenetic correlation parameter calculated from phyloglm. Bold *P*-values are significant (*P* < 0.05) without corrections for multiple comparisons. None of the analyses were significant (*P* < 0.05) after correcting for false discovery rate (the bold values were ∼*P* = 0.1) or an even more conservative Bonferroni-corrected *P*-critical value set to 0.0038.

## DISCUSSION

We begin with three fundamental limitations of our data and the inferences drawn about Takotsubo Cardiomyopathy. Like the results of other comparative and phylogenetic analyses (and indeed like the results of meta-analyses), our results and conclusions are limited by the existing data. The prevalence of CM in animals being captured for research and management purposes is probably greatly underestimated for several reasons, even in the very well-studied ungulates. First, even when efforts are made to quantify and describe mortality of animals being captured, the carcasses are rarely subjected to a thorough necropsy that could result in a diagnosis [46]. Without a thorough necropsy, the occurrence of CM can only be presumed, and other causes of death are included in the observed mortality rate. Second, an unknown and unobserved proportion of CM deaths occur after the captured animals are released and the carcasses may not be recovered. Third, CM is probably a continuum of effects, possibly affecting a majority, or even all animals that are captured, with an unknown portion of those affected to the point of showing signs recognizable as CM. Finally, impairment following capture and processing may be attributed to other causes.

We must also note that CM is likely to be a composite trait and may not be the same as Takotsubo cardiomyopathy. CM syndromes typically affect muscle tissue diffusely with affects noted in both skeletal and cardiac muscle whereas Takotsubo CM specifically refers to a stress-induced cardiac muscle effects only. Both syndromes are triggered by emotional or physical stress and present with increased amounts of catecholamines and creatinine kinase levels [34, 47]. CM is characterized further by metabolic acidosis caused by a lactic acid buildup from muscle degeneration [48]. Takotsubo is mainly described by a bulge in the left ventricle, following the surge of catecholamines, which causes left ventricular dysfunction [49]. Despite the differences, CM has pathophysiological similarities to Takotsubo that permits us to hypothesize CM as a model.

We also recognize that none of the relationships we explored were significant after corrections for multiple comparisons. There is a debate about whether and how to correct for multiple comparisons [50]. However, because some of the independent variables were correlated or provided somewhat redundant measures of some important aspect of life history variation, it is not clear what the appropriate correction for multiple comparisons should be. Ultimately, given the nature of these data, and the analyses, we feel that these results should be interpreted in an exploratory way.

Despite these potential shortcomings, the current results are intriguing. We focus on interpreting the phyloglm results because of the significant phylogenetic signal in many of these traits. Susceptibility to CM in the well-studied ungulates may be an unavoidable consequence of adaptations to reduce predation risk. Increased running speed, for example, requires an explosive autonomic (sympathetic) response to threat. Those species that are able to run fast are more susceptible to CM, possibly because of the autonomic response to threats. Sociality and larger brains, also adaptations associated with reduced predation risk are associated with more complex autonomic systems. Indeed, more social species, including ungulates, may have larger brains [51]. Moreover, longer-lived species are composed of individuals that have successfully avoided predation. These species seem to be more likely to be susceptible to CM. This might be because they are more likely to have encounters with predators throughout their lives. Alternatively, it might be because longevity is part of a syndrome of life history traits that predisposes species towards having a susceptibility to CM.

Not all genera have reports of CM and this provides the opportunity to identify particularly susceptible (or not susceptible) animal groups or ecological scenarios/constraints that are associated with this syndrome. By doing so we may comparatively examine the mechanisms that underlie vulnerability to fear induced sudden death across species. Future studies are clearly warranted here.

Additionally, it is possible that the processes that make a species susceptible to CM which we suggest may be part of a syndrome to escape predators, may be adaptive in high predation risk environments and more costly in lower predation risk environments. Future intraspecific studies are required that would first document variation within a species in the propensity to experience CM, and then to quantify CM susceptibility frequency in different habitats with different predation risk. Because latitude [52] and being found on an island [53] is associated with predation risk, future empirical studies may capitalize on these sources of natural variation to evaluate the adaptive hypothesis.

Aside from identifying potential future research targets, these phylogenetic results suggest that CM may be an unavoidable consequence of being social and long-lived, at least in ungulates. To translate this comparative finding into meaningful health recommendations requires an additional assumption: the factors that explain between species variation are the same as those that explain within species variation. There are a number of reasons why this may not be so (e.g. the range of variation at the intraspecific level is less than that at the interspecific level), but interspecific comparative results often allow the development of intraspecific testable hypotheses. Future studies of human data sets could help determine whether similar factors explain human susceptibility to Takotsubo cardiomyopathy. And, if so, future interventions might capitalize on manipulating life history trajectories to reduce the likelihood of experiencing Takotsubo cardiomyopathy during highly stressful episodes.

While highly speculative, such interventions may capitalize on our understanding that animals exposed to early stressful experiences have lasting effects [54, 55]. For instance, in mammals individuals who experience early direct exposure to predators, or more indirect maternal effects associated with living in a high-risk environment, follow a very different life history trajectory than individuals who grow up in relatively safer environments. If such early exposures prepare animals to escape quickly, for example by reallocating energy towards developing fast twitch muscle fibres or having a highly active sympathetic nervous system, then they might increase the susceptibility to CM. If so, it the benefits of reducing maternal stress and early childhood stresses may have lasting impacts in terms of reducing the likelihood of later being susceptible to Takotsubo cardiomyopathy.

### Clinical implications

A diagnosis of Takotsubo cardiomyopathy is made when extreme stress, either psychological or physiological, precedes an elevation of plasma catecholamines and an acute and unique form of left ventricular dysfunction (heart failure). Intense emotional states including fear and grief were initially identified as precipitants to the syndrome in humans. Recently, however, there has been increased awareness of the potential for physiological stresses such as sepsis, post-operative state, and myocardial infarction to precipitate the syndrome as well [56, 57].

It is notable that this unique form of cardiomyopathy occurs in individuals who seem to be experiencing the highest levels of acute stress. Of course, the vast majority of human patients experiencing high levels of either psychological or physiological stress do not develop Takotsubo cardiomyopathy. The fact that some do, however, suggests that vulnerability to the syndrome is linked to the tendency of an individual to mount a robust and intense catecholamine response to stress.

That the vulnerability to Takotsubo cardiomyopathy varies among individuals is not surprising as most medical syndromes have multiple contributing causes including variation in the genetic background and a variety of environmental factors. However, the variability in susceptibility to Takotsubo cardiomyopathy among individuals point, at least in part, to a diversity in how intense an individual’s autonomic response to stress. Recognition of differential vulnerability to Takotsubo cardiomyopathy between individual humans leads to the connected but expanded comparative questions: what are the differential vulnerabilities across species and what are the mechanisms that underlie these differences?

Identifying the unique characteristics of animal species with and without vulnerability to peracute CM could create a pathway for human investigators to study vulnerability in humans. For example, comparing the functional integrity of sympathetic nerve terminals or the endogenous catecholamine levels in myocardial interstitial fluid across species with differential vulnerability could explain why one human being exposed to the same objective level of stress is vulnerable while another is not. This, then, could point to pharmacological or device driven therapeutics for these individuals. Furthermore, the ability to identify at risk populations could promote prevention strategies for vulnerable populations. Finally, the concept of evolutionarily framed life history traits has little penetration in the world of human medical thought and investigation. The introduction of this perspective could spark novel hypotheses pointing to population-based public health oriented strategies for Takotsubo cardiomyopathy and related syndromes.

## FUNDING

D.T.B. is supported by NSF DEB-1119660; J.B. was both a GAANN Fellow and a Fulbright Fellow while conducting this work; M.E.A. is supported by the NSF.

**Conflict of interest**: None declared.
